# FISH-Based Analysis of Clonally Derived CHO Cell Populations Reveals High Probability for Transgene Integration in a Terminal Region of Chromosome 1 (1q13)

**DOI:** 10.1371/journal.pone.0163893

**Published:** 2016-09-29

**Authors:** Shengwei Li, Xiaoping Gao, Rui Peng, Sheng Zhang, Wei Fu, Fangdong Zou

**Affiliations:** 1 Key Laboratory of Bio-resources and Eco-environment (Ministry of Education), College of Life Sciences, Sichuan University, Chengdu, Sichuan 610064, PR China; 2 Chengdu Bio-Tech Co. Ltd., Chengdu, Sichuan 610041, PR China; 3 Department of Physiology, West China School of Preclinical and Forensic Medicine, Sichuan University, Chengdu, Sichuan 610064, PR China; Nazarbayev University, KAZAKHSTAN

## Abstract

A basic goal in the development of recombinant proteins is the generation of cell lines that express the desired protein stably over many generations. Here, we constructed engineered Chinese hamster ovary cell lines (CHO-S) with a pCHO-hVR1 vector that carried an extracellular domain of a VEGF receptor (VR) fusion gene. Forty-five clones with high hVR1 expression were selected for karyotype analysis. Using fluorescence *in situ* hybridization (FISH) and G-banding, we found that pCHO-hVR1 was integrated into three chromosomes, including chromosomes 1, Z3 and Z4. Four clones were selected to evaluate their productivity under non-fed, non-optimized shake flask conditions. The results showed that clones 1 and 2 with integration sites on chromosome 1 revealed high levels of hVR1 products (shake flask of approximately 800 mg/L), whereas clones 3 and 4 with integration sites on chromosomes Z3 or Z4 had lower levels of hVR1 products. Furthermore, clones 1 and 2 maintained their productivity stabilities over a continuous period of 80 generations, and clones 3 and 4 showed significant declines in their productivities in the presence of selection pressure. Finally, pCHO-hVR1 localized to the same region at chromosome 1q13, the telomere region of normal chromosome 1. In this study, these results demonstrate that the integration of exogenous hVR1 gene on chromosome 1, band q13, may create a high protein-producing CHO-S cell line, suggesting that chromosome 1q13 may contain a useful target site for the high expression of exogenous protein. This study shows that the integration into the target site of chromosome 1q13 may avoid the problems of random integration that cause gene silencing or also overcome position effects, facilitating exogenous gene expression in CHO-S cells.

## Introduction

Biotechnological advances have provided a number of attractive options for the manufacturing of protein pharmaceuticals. However, the generation of stable, high-producing cell lines remains a major challenge in the early stages of the manufacturing process. Currently, numerous methods are available to predict high protein productivity and stability of recombinant protein expression. For example, dihydrofolate reductase (DHFR) amplifies the copies of an exogenous gene to increase the productivity of recombinant proteins [1–3]. However, high gene copy numbers do not always result in high protein productivity [4–8]. The loss of gene copies is only partially responsible for decreased antibody productivity. Chromosomal aberrations including dicentrics, rings, and extremely long chromosomes may be the primary reason underlying protein production instability [9, 10]. Recent observations [11] have found that a high heavy chain (HC) and light chain (LC) gene copy number did not result in high productivity, which was consistent with previous reports [12].

In contrast to the gene copy number, the chromosomal integration of an exogenous gene appears to be more considered. Due to the random nature of an integration event, several sites may be incapable of supporting the transcription of the exogenous gene or the expression level is greatly influenced by the effect of the genetic environment at the gene locus (position effects) [9, 10]. Analysis of a broad range of recombinant cell lines and their integration loci found that the majority of highly producing cell lines have an insertion locus on large chromosomes [4, 6]. The fragile site and the telomeric region have been proposed to be the preferred integration site of the stable transfectant [13, 14]. However, others have suggested that gene integration at known fragile sites can lead to genomic instability, thus affecting the expression of targeted genes [15].

Chinese hamster ovary (CHO) cell lines, owing to their superiority for post-translational modifications of folding, assembly and glycosylation, are widely used to produce therapeutic recombinant proteins including monoclonal antibodies and vaccines. Several variants of CHO cell lines, CHO-K1, CHO-DG44 and CHO-S, have been widely used in the biopharmaceutical industry. Cytogenetic analysis shows that chromosomal rearrangement is a characteristic feature in CHO cell lines [16–18]. The CHO-K1 line, in contrast to the 22 chromosomes in diploid CHO cells, with 21 chromosomes, has only eight chromosomes that appear equivalent to those of the normal Chinese hamster chromosomes. In addition, thirteen chromosomes are designated as Z group chromosomes that contain deletions, reciprocal and nonreciprocal translocations and pericentric inversions [19, 20]. Chromosomal aberrations and the instability of recombinant protein expression have been previously observed [4, 9, 10]. More specifically, several of the locations where exogenous genes are integrated are incapable of supporting transcriptional events or the expression levels of exogenous genes can be influenced by the local genetic environment at the gene locus. Therefore, we sought to determine which chromosomal regions may be favorable in terms of expression and transgene stability. To achieve this goal, we generated a series of recombinant CHO-S clones and identified the integration sites of an exogenous gene. Thereafter, we selected several clones to compare their recombinant protein production levels and stability. We demonstrate that CHO-S clones with the exogenous gene located at chromosome 1q13 can generate a high yield of recombinant protein and stable recombinant cell lines.

## Materials and Methods

### Plasmid Construction

The pCHO-hVR1 plasmid encoding a recombinant protein hVR1 (VEGF-Trap_R1R2_/Fc) [21] against VEGF was constructed by inserting a 1.5 kilobase (kb) *Avr*II-*BstZ17*I hVR1 expression fragment into the pCHO1.0 vector (A13369, Life Technologies) that contains the puromycin resistance gene, which confers resistance to the antibiotic puromycin. The pCHO-hVR1 expression vector contained the hVR1 sequence, which was under the control of the EF2/cytomegalovirus (CMV) hybrid promoter and initiates the transcription of the recombinant protein. Furthermore, the coding sequence for the bacterial kanamycin gene was under the control of the SV40 early promoter. Both the dihydrofolate reductase (DHFR) gene and puromycin resistance gene were under the control of the phosphoglycerate kinase (PGK) promoter for the selection of transfected cells using methotrexate (MTX) and puromycin [22–24]. CHO-S^™^ cells were used as the host cell line for recombinant protein expression.

### Cell Culture and Screening of Recombinant Clones

Pre-adapted parental CHO-S cells (Life Technologies) were cultured in suspension in a protein-free CD FortiCHO^™^ medium (Life Technologies) supplemented with 8 mM L-glutamine and anti-clumping agent (Life Technologies) at a 1:100 dilution.

For the generation of recombinant protein-expressing cell lines, 1×10^7^ CHO-S cells were transfected by electroporation in the presence of linearized pCHO-hVR1 using Nucleofector technology (Amaxa, Gaithersburg, MD) and the Cell line Nucleofector kit V (Lonza) according to the manufacturer’s instructions. To generate a pool of stable transfectants, the transfected cells were incubated with 10 μg/mL puromycin and 100 nM MTX for selection phase 1. For selection phase 2, puromycin was added to a concentration of 30 μg/mL, and MTX was added to a concentration of 500 nM. For the final selection phase 3, puromycin was added to a concentration of 50 μg/mL, and MTX was added to a concentration of 1000 nM. All the selection phases were completed when viability exceeded 85%, and the viable cell density exceeded 1×10^6^ viable cells/mL. The transfected cells were separated into 6-well plates at 500 cells per well and were allowed to grow in semi-solid medium containing methylcellulose (Molecular Divices) for 10 days. A labeled antibody (Clone Detect, Human IgG Specific, Fluorecein, Molecular Devices) was used at a concentration of 100 U/mL to monitor hVR1 expression. Single colonies with fluorescent signals were picked into the 96-well plates using the Clonpix system. To select the best producing clones to monitor the improvement in protein productivity, different clones and cell pools were screened for their productivity using the ForteBio Octet System (Pall). Finally, the high yield single clones were selected and expanded by a 6-well cell culture cluster in an incubation shaker (Inforce HT, Multitron Pro).

For long-term cultures, several representative clones selected from high yield single clones were cultured in a shake flask containing 15 mL of CD FortiCHO ^™^ medium in the presence or absence of the final concentrations of MTX and puromycin. The initial cell concentration in each culture was adjusted to approximately 3×10^5^ cells/mL. Cell concentration and viability were determined using the Count Star^™^ cell counter. Culture supernatant samples for protein assays underwent several passages and were kept frozen at—80°C.

### Chromosome Preparation and G-banding

All the selected recombinant cells and non-recombinant CHO-S cells were used for chromosome preparation. The cells (1×10^6^) were treated with colchicine (0.5 μg/ml) at 37°C for 3 hours. The cells were then exposed to a hypotonic shock with 37.5 mM KCl for 20 min, fixed with 25% acetic acid and 75% methanol and spread onto a glass microscope slide. After a few days, the chromosomes of high yield single clones and normal CHO-S cells were banded using Trypsin-Giemsa (G-banding) techniques. The metaphases were exposed for 10–20 s to trypsin (1% in EDTA), washed with 150 mM NaCl, and dried at room temperature. The slides were then stained with Giemsa solution (2% in PBS) for 8 min for visualization of the chromosomal G-band pattern. The chromosomes prepared from CHO-S cells were sorted and identified by comparisons with the CHO G-band karyotype [19, 25, 26], whereas the chromosomes prepared from recombinant CHO-S cells were used for fluorescence in situ hybridization. All the karyotypes were analyzed using the ZEISS Imager 2 system and Metasystems Ikaros software.

### Fluorescence *in Situ* Hybridization

DNA fragments were amplified from the pCHO-hVR1 plasmid and were used for probe preparation. Gene-amplified primers were designed from GENEWIZ, Inc. (P1: 5’-GACAGTAGAAAGGGCTTCATC-3’; P2: 5’-CTTCCACCAGAGATTCCATGCCAC -3’). The amplified DNA was labeled with the Random Primed DNA Labeling Kit (Roche 1104760001) with Biotin-16-dUTP (Roche 11093070910). The probe length was confirmed to be approximately 100–500 bp.

FISH was performed as previously described [13]. All the chromosome slides were denatured in 70% formamide/2×SSC at 75°C for 2 minutes. In parallel, the probe was denatured at 78°C for 10 minutes and applied to the slides. Hybridization was conducted overnight at 37°C for 16 hours. The slides were washed three times at 43°C in 50% formamide/2×SSC and three times at 43°C in 2×SSC. The probe was visualized by subsequent incubation with fluorescein isothiocyanate-labeled streptavidin (FITC Streptavidin) (BioLegend 405201). The chromosomes were counterstained with 4', 6-diamidino-2-phenylindole (DAPI)-containing antifade solution (Millipore S7113), and the FISH images were captured using the ZEISS Imager 2 System and Ikaros software.

### Determination of Productivity

To determine the productivity of the representative clones and stable pools, the cells were cultured in a shake flask and passaged every 4 days. When the cells were extended to 5 passages (P5), 30 passages (P30) and 80 passages (P80), the shake flask cultures were monitored, and culture supernatant samples were cultured on days 3, 5, 7, 9, 11, 13 and were analyzed for secreted protein. The cell concentration was determined in a CountStar^™^ Cell Counter (Inno-Alliance Biotech Coulter), and the secreted cell supernatant product concentration was quantified using a ForteBio Octet Platform. The secreted protein concentration was quantified for the hIgG Fc part of the fusion protein. Based on the platform, the Anti-Human IgG Fc biosensor was used to capture the fusion protein. A 1-ml sample from each culture was centrifuged at 1000 rpm for 4 min. The supernatant samples were stored at -20°C before the assay was conducted. Affinity-purified hVR1 was used as a standard to establish a standard curve. The supernatant concentrations were calculated from a non-linear fit of the data using the Octet software v.6.1. For each sample, the assay was performed in triplicate.

### Analysis of Gene Copy Numbers

During various periods of cell culturing, the cells were collected to extract genomic DNA. Genomic DNA was isolated from 6×10^6^ cells using the QIAamp DNA Mini Kit (QIAGEN) according to the manufacturer’s instructions. The concentration and quality of the DNA were determined using agarose gel electrophoresis and UV spectrophotometry at 260 and 280 nm (MD-SpectraMax M5). Relative gene copy numbers were determined using real-time quantitative PCR (RT qPCR). hVR1 and β-actin primers (Table 1) to specifically amplify the respective target DNA sequences were designed using Primer Premier software. RT qPCR was performed by employing the Opticon 2 Detection System in conjunction with the SYBR Premix Ex Taq^™^ (TaKaRa) following the manufacturer’s instructions. For each PCR reaction sample, 10 ng genomic DNA (for the analysis of gene copy numbers) was mixed with 10 μL SYBR Premix Ex Taq^™^, 5 mM primer mix, and nuclease-free water for a final total reaction volume of 20 μL, which was then split into duplicates. The following thermal cycling parameters were applied: 30 s at 95°C (initial denaturation), followed by 40 cycles of 5 s at 95°C and 40 s at 55°C. The pCHO-hVR1 and pMD-β-actin plasmids were constructed to establish a standard curve. The data were analyzed using the Opticon Monitor 3 Software. Each PCR reaction was performed in triplicate to compensate for potential pipetting errors.

**Table 1:** Primer pairs and their production length.

Name	Primer sequence	Amplicon size (bp)
hVR1-F	5’-GTGGAGTGGGAGAGCAATGG-3’	170
hVR1-R	5’-TGCTGCCACCTGCTCTTGT-3’	
β-actin-F	5’-TCTATGAGGGCTACGCTCTCC -3’	102
β-actin-R	5’-ACGCTCGGTCAGGATCTTCA -3’	

## Results

### Generation of recombinant CHO-S cell clones

**Fig 1:**
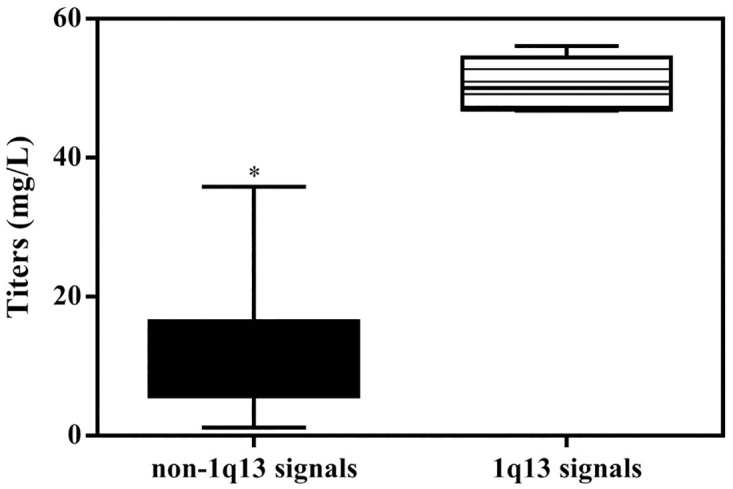
The productivity of recombinant clones. *compared with 1q13 signals group, p<0.05.

To generate recombinant cell clones, we introduced the pCHO-hVR1 vector into CHO-S cells. The transfected cells were screened in semisolid media with MTX/Puromycin, and then expanded in liquid culture medium. Finally, we screened about 500 clones, and ELISA assay was employed to evaluate the expression of hVR1 of these clones. As shown in Fig 1, a wide expression pattern of the hVR1 transgene was obtained in these clones, in which six clones showed the highest hVR1 expression. Forty-five clones with high levels of hVR1 expression were selected for further studies.

### Chromosomal Distribution and Location of pCHO-hVR1

**Fig 2:**
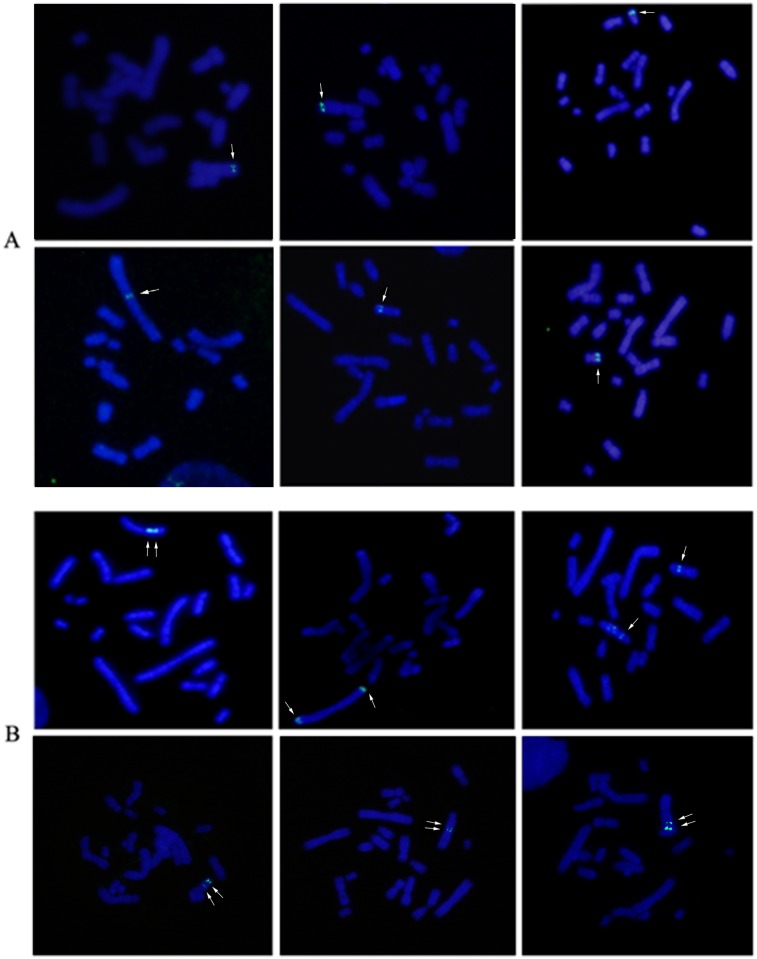
FISH chromosomal integration patterns. (A) Representative clones with single signal distribution in one chromosome, and (B) multi-signals in one or two chromosomes.

**Fig 3:**
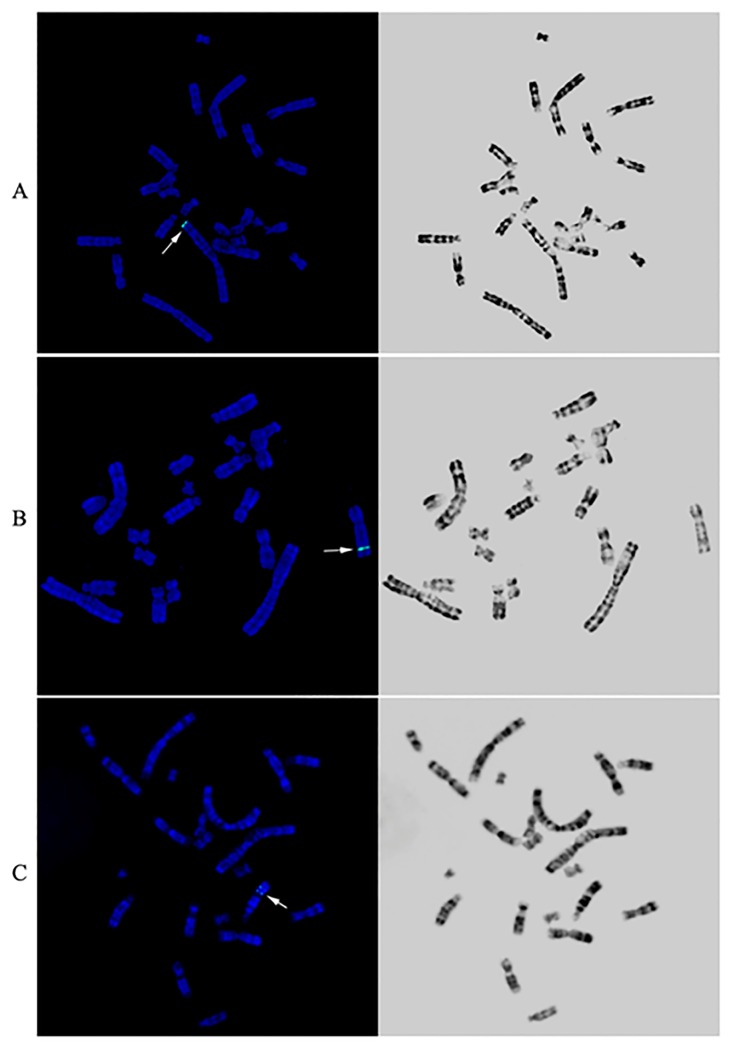
Three representative clones with hVR1 integration feature. Arrows indicate hVR1 genes located on the long arm of chromosome 1 (A), the long arm of chromosome Z3 (B) and the long arm of chromosome Z4 (C).

**Fig 4:**
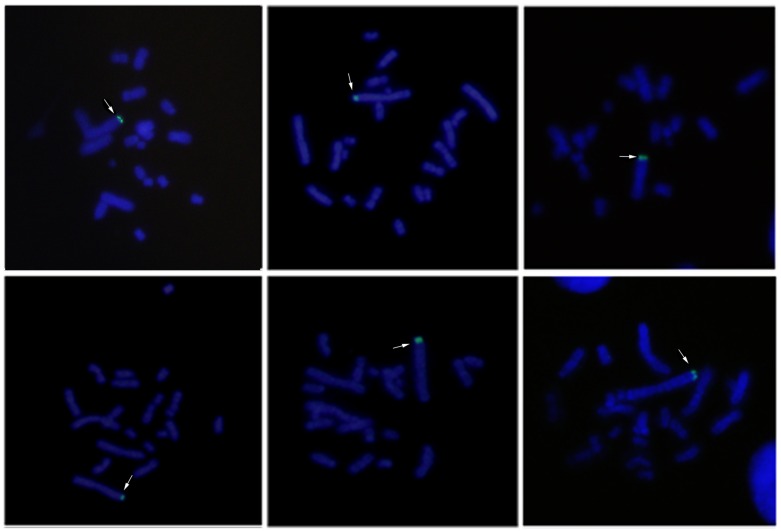
FISH signals at the same ends of the long arm of chromosome 1.

To determine the chromosomal distribution of pCHO-hVR1 integration, we generated 45 FISH hybridization clones and analyzed 50–100 metaphase cells of each clone. Fig 2 shows the distribution of specific FISH signals (labeled in green) on chromosomes of the representative clones, and either a single signal at one chromosome (Fig 2A) or multiple signals at one or two chromosomes were observed (Fig 2B). These results indicated that pCHO-hVR1 was successfully integrated into the host genome. To determine the characteristic of pCHO-hVR1 integration into different chromosomes, a combination of FISH with G-banding was performed in the 45 clones. More than 60% of the clones showed FISH signals distributed on three chromosomes, including chromosomes 1 (11 clones), Z3(10 clones) and Z4 (8 clones). The remaining 16 clones showed FISH signals distributed on all other chromosomes at a very low counting frequency. The single integration site was found to be the primary feature in normal chromosome 1, in which the specific signals were distributed at the ends of the long arm of chromosome 1 (Fig 3A). In addition, the single signal or multi-signal distributions were observed in the long arms of chromosomes Z3 and Z4 (Fig 3B and 3C), respectively. Similar integration features were also observed in other normal chromosomes and the Z group chromosomes. Notably, six clones with the highest hVR1 expression (Fig 1) had the FISH signals at similar ends of the long arm of chromosome 1 (Fig 4).

**Fig 5:**
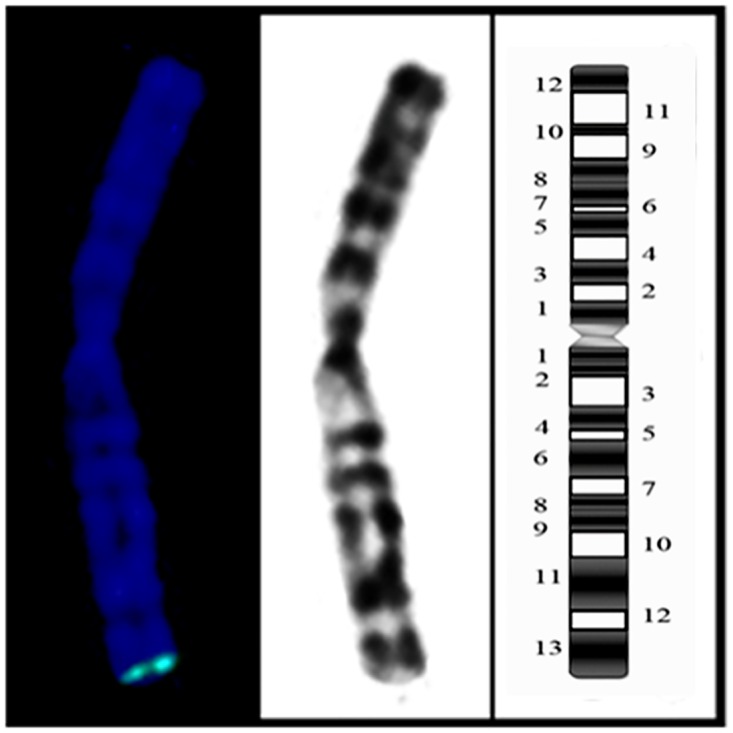
Chromosomal location 1q13.

Six clones with the high hVR1 expression (Fig 1) showed the same integration site on the terminal region of the long arm of chromosome 1, which led us to the identification of the pCHO-hVR1 target site in chromosome 1. Using a combination of FISH with G-banding, pCHO-hVR1 was localized at chromosome 1q13 (Fig 5). The result suggested that 1q13 can be a target area, at least in CHO-S cell line, that can support the high transcription of the hVR1 gene or high expression of recombinant proteins.

### Determination of Productivity

**Fig 6:**
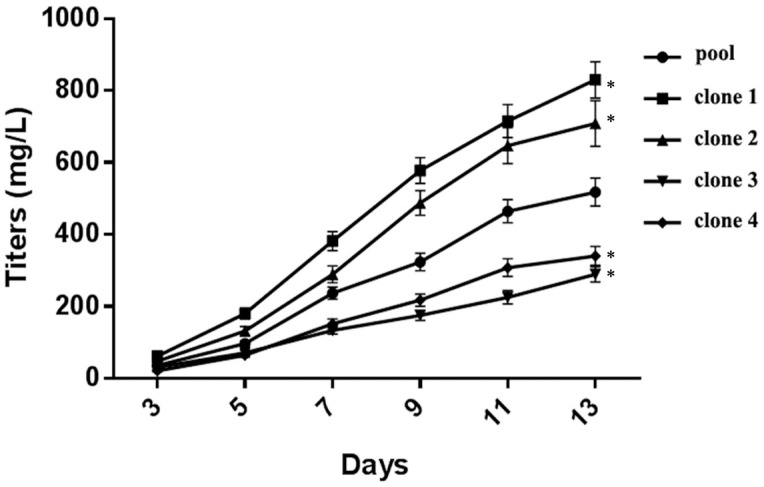
Comparison of the productivity in four clones and the pooled cells. *compared with pool cell (13 days), p<0.05.

To determine whether high and stable productivity may be correlated with the integration site on chromosome 1, two high protein-producing clones with the same integration sites at chromosome 1 (clones 1 and 2) were selected for their hVR1 production levels under non-fed, non-optimized shake flask conditions. Simultaneously, two other higher protein-producing clones, clones 3 and 4, with different integration sites at chromosomes Z3 and Z4, were also selected as a productivity comparison. As shown in Fig 6, clones 1 and 2 showed higher protein expression than clones 3 and 4 on day eleven. On day thirteen, the protein expression of clone 1 reached the highest level with titers of approximately 800 mg/L, which was 2.3-fold higher than clone 4 and 3.6-fold higher than clone 3. Similar results were obtained with clone 2, which showed high titer levels of approximately 700 mg/L on day thirteen. Although both clones 3 and 4 provided similar protein levels (approximately 50 mg/L) with clones 1 and 2 on day three, the increasing titers in clone 1 (or clone 2) while clone 3 (or clone 4) did not show the same rate of titer increase during the subsequent culture for 7 days. The four clones were isolated from the same pooled line, and clones 3 and 4 showed titers even below the pooled cell titers, suggesting that the two clones, despite their high baseline levels, did not result in the sustained duration of high hVR1-producing. Whereas the clones with the integration sites at chromosome 1 were sustained VR1 expression, suggesting the clones has potential to become high-producing cell lines.

**Fig 7:**
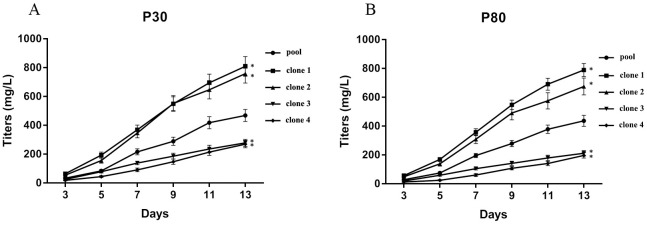
The productivity of four clones during long-term culturing. (a) P30: Passage 30, (b) P80: Passage 80. *compared with pool cell (13 days), p<0.05.

We further determined whether the highly producing CHO-S cell lines can maintain their stable productivity after continuous generations. The productivity of four clones from continuous culturing over 30 and 80 generations was assessed under non-fed, non-optimized shake flask conditions. As shown in Fig 7A and 7B, clones 1 and 2 maintained the highest hVR1 expression, significantly higher than clones 3 and 4 during either 30 passages or 80 passages. These results were similar to the data obtained from 5 passages (Fig 6), indicating that clones 1 and 2 maintained their productivity stability in long-term cultures, even over a continuous period of 80 generations. Thus, the highly producing CHO-S cell line appears to be associated with pCHO-hVR1 integration at chromosome 1. Conversely, integration into chromosomes Z3 and Z4 did not result in the generation of highly producing CHO-S cell lines, although they shared similar high integration frequencies.

**Fig 8:**
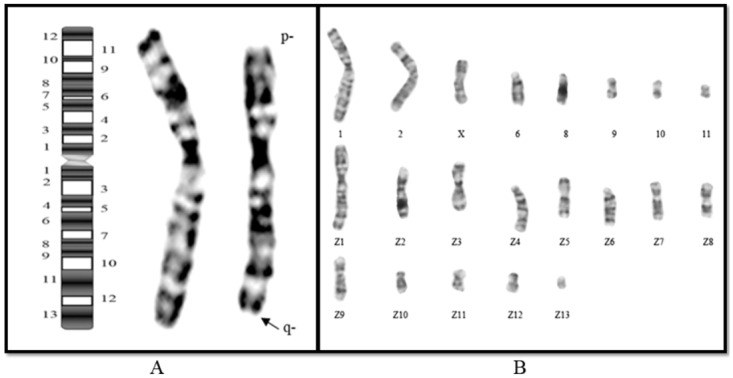
Trypsin-Giemsa banded karyotype from CHO-S metaphase cells. (A) CHO-S karyotype. (B) G-banded normal chromosome 1 (left) and chromosome Z1 (right). A new terminal deletion was found on the long arm of chromosome Z1.

In all 45 clones, we never observed the integration of pCHO-hVR1 on chromosome Z1, one of a pair of chromosomes 1. We analyzed the chromosomal karyotype of the CHO-S cell line. As shown in Fig 8, approximately 80% of CHO-S cells contain 21 chromosomes, in which 8 are normal and 13 are rearranged Z group chromosomes, with a modal karyotype (Fig 8A) similar to the CHO-K1 cell line described by Deaven [19]. However, a minor difference exists between these cell lines. Specifically, in CHO-S cells, the difference contains a terminal deletion from the long arm of the Z1 chromosome. However, we found that almost all the CHO-S cells that had a chromosomal terminal deletion occurred at Z1q12, thereby resulting in the loss of Z1q12-13 region (Fig 8B). This finding may help explain why we never observed the integration of pCHO-hVR1 on chromosome Z1.

### Gene copy number analysis

We finally determined the gene copy number of the cell clones. The absolute amount of pCHO-hVR1 was analyzed in samples containing 10 ng of genomic DNA, enabling gene copy number quantification even in cell lines without gene amplification. We examined the copy number of four clones between 5 passages and 80 passages. Gene copy numbers per cell are presented in Table 2. No loss of gene copy numbers was observed in clones 1 and 2; however, clones 3 and 4 lost one gene copy number during the long-term culturing. This study demonstrates that the four clones are different clones, especially clones 1 and 2, which have similar 1q13 integration regions.

**Table 2:** Estimation of the hVR1 gene copy number.

Clones	P5		P80	
	hVR1/β-actin	hVR1 copy numbers	hVR1/β-actin	hVR1 copy numbers
Clone 1	2.76	3	3.22	3
Clone 2	2.4	2	2.35	2
Clone 3	2.9	3	2.04	2
Clone 4	4.46	4	3.05	3

## Discussion

The generation of highly producing cell lines expressing exogenous genes facilitates the development of therapeutic recombinant proteins [27, 28]. Although recombinant protein expression can be easily achieved by co-amplifying an exogenous gene alongside a selectable marker, such as dihydrofolate reductase, high level expression of recombinant protein is often affected by the integration sites and their genetic environment (position effects) in the chromosomes of host cells [29]. Therefore, the necessity of obtaining a desirable integration site for recombinant protein expression has been considered as a result of the generation of high-producing CHO cell lines, especially as a result of the generation of highly producing CHO cell lines by homologous recombination.

We generated a series of recombinant clones and characterized chromosomal sites where pCHO-hVR1 integrated in a random manner. Most recombinant clones revealed that pCHO-hVR1 was integrated into three chromosomes, chromosome 1, chromosome Z3 and Z4. However, only the clones with the integration site in the terminal region of the long arm of chromosome 1 had high and stable productivity of recombinant protein. Other clones with integration sites on chromosome Z3 and Z4 had lower productivities even below the pooled cell levels. Using combination fluorescence *in situ* hybridization and G-banding, pCHO-hVR1 was located at chromosome 1q13. Surprisingly, the clones (Fig 1) with the highest hVR1 expression in the early process were identified to have the same integration sites at the telomeric region of chromosome 1q, suggesting that the telomeric region of chromosome 1 may be the preferred site for exogenous gene integration. In addition, we obtained similar results by using another plasmid which encodes a recombinant GLP-1 protein (S1 Fig and S1 Table).

Early studies [14] have shown that telomere-type clones, in which the exogenous gene is located near the telomeric region, were found to have a specific hGM-CSF production rate, approximately 6 times higher than the rate of other type of clones. Our study also provided evidence that the telomeric region at chromosome 1 appears to be a desired site for exogenous gene integration. However, not all telomere-type clones were found to be stable and productive. For example, a few clones, in which the exogenous gene was located in the telomeric region of other chromosomes such as the Z group chromosome, were eliminated in the early screening stage due to low protein expression. Chromosomal rearrangement is a characteristic feature of CHO cell lines such as in the CHO-K1 line, which in contrast to the 22 chromosomes in diploid CHO cell, has 21 chromosomes. Furthermore, of these 21 chromosomes, only eight appear equivalent to the normal Chinese hamster chromosomes, and thirteen are designated as Z group chromosomes that contain deletions, reciprocal and nonreciprocal translocations and pericentric inversions [19, 20]. The CHO-S cell line is derived from the CHO-Toronto cell line and is apparently derived from a population of cells that gave rise eventually to the CHO-K1 cell line [30]. As shown in Fig 8, approximately 80% of CHO-S cells contained 21 chromosomes, in which 8 were normal and 13 were rearranged Z group chromosomes, having modal karyotype (Fig 8A) similar to the CHO-K1 cell line described by Deaven [19], with only a minor difference. Several of the telomeric regions, especially in rearranged chromosomes, did not support the transcription of exogenous gene and protein expression, suggesting that the genetic environment at the telomeric region of the Z group chromosome may change because of chromosomal rearrangements. We support the conclusion made by Yoshikawa [14] suggesting that telomere-type clones have high and stable productivity of recombinant protein, but we are inclined to consider that the integration site in telomeric regions at normal chromosomes may be influenced by the effects of the local genetic environment. Additionally, evidence suggests that chromosomal aberrations, including dicentrics, rings, and extremely long chromosomes, may be the main reason underlying the productivity instability of recombinant monoclonal antibodies [9, 10].

Interestingly, in our study, we never observed the integration of pCHO-hVR1 on chromosome Z1, one of the chromosome 1 pairs. Thereafter, we found that a terminal deletion occurred in the long arm of the Z1 chromosome, which may explain why we never observed the integration of pCHO-hVR1 on chromosome Z1 in CHO-S cells. This finding also suggests that the loss of the telomeric region of 1q13 may lead to low frequency integration in CHO-S cells, in contrast to other CHO lines, such as CHO K1 and DG44. In addition, we utilized qPCR to demonstrate that clones 1 and 2 are different clones that have similar 1q13 integration regions that can express proteins efficiently. Our studies revealed the advantage of the integration into chromosome 1q13 to overcome the position effects [31] and to facilitate exogenous gene expression in CHO-S cells, avoiding gene silencing based on random integration.

In conclusion, using a combination of fluorescence *in situ* hybridization and G-banding, we identified a specific site, chromosome 1q13, which supports stable integration of the hVR1 gene and promotes its strong expression in the CHO-S cell line. Chromosome 1q13 may contain a preferred integration site of exogenous genes for the screening of recombinant clones in CHO-S cells and may be considered as the chromosomal landing pad [32] for homologous recombination. In future studies, site-integrated cell lines can be constructed by genetic engineering methods, and these cell lines can be used to target the integration of exogenous genes to chromosome-specific loci, which can save screening time and create stable expression cell lines.

## Supporting Information

S1 Fig Six clones with GLP-1 integration feature. Arrows indicate GLP-1 genes located on different chromosomes, GLP-1 genes located on the 1q13 (E), GLP-1 genes located on the non-1q13 (A, B, C, D, F).
(ZIP)

S1 Table Comparison of the Glp-1 productivity of six clones. (ZIP)
