# Supplementary figures and images for: FISH-Based Analysis of Clonally Derived CHO Cell Populations Reveals High Probability for Transgene Integration in a Terminal Region of Chromosome 1 (1q13)
Source: PLoS One. 2016 Sep 29;11(9):e0163893. doi: 10.1371/journal.pone.0163893 (PMC5042417; doi:10.1371/journal.pone.0163893)

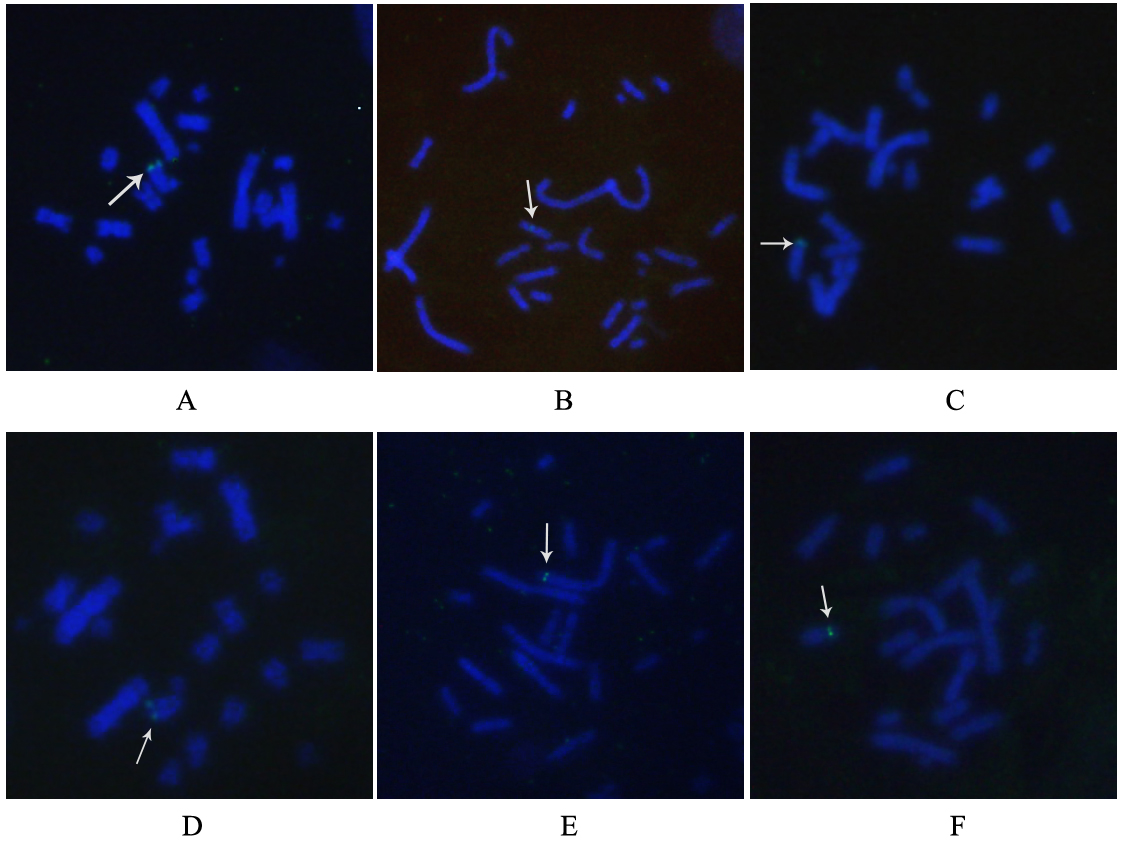

Supplement: S1 Fig — Arrows indicate GLP-1 genes located on different chromosomes, GLP-1 genes located on the 1q13 (E), GLP-1 genes located on the non-1q13 (A, B, C, D, F). (ZIP) [file pone.0163893.s001.zip › S1 Fig/S1 Fig.tif]
