# Supplementary material for: FISH-Based Analysis of Clonally Derived CHO Cell Populations Reveals High Probability for Transgene Integration in a Terminal Region of Chromosome 1 (1q13)
Source: PLoS One. 2016 Sep 29;11(9):e0163893. doi: 10.1371/journal.pone.0163893 (PMC5042417; doi:10.1371/journal.pone.0163893)
Supplement: S1 Table — (ZIP) [file pone.0163893.s002.zip › S1 Table/S1 Table.docx]

**S1 Table. Comparison of the Glp-1-Fc productivity of six clones.**

| Clones  (Passages 5) | Concentration（μg/ml） |
| --- | --- |
| Clone A | 5.42±1.24 |
| Clone B | 2.88±0.86 |
| Clone C | 9.74±2.14 |
| Clone D | 23.70±3.18 |
| Clone E | 71.95±5.24 |
| Clone F | 39.10±4.39 |
